# Supplementary material for: Towards n-type conductivity in hexagonal boron nitride
Source: Nat Commun. 2022 Jun 3;13:3109. doi: 10.1038/s41467-022-30762-1 (PMC9166779; doi:10.1038/s41467-022-30762-1)
Supplement: Supplementary file 1 — Supplementary Information [file 41467_2022_30762_MOESM1_ESM.pdf]

Supplementary Information:

Towards *n*-Type Conductivity in Hexagonal Boron Nitride

Shiqiang Lu[1,3], Peng Shen[1,3], Hongye Zhang[1], Guozhen Liu[1], Bin Guo[1], Yehang Cai[1], Han Chen[1], Feiya Xu[1], Tongchang Zheng[2], Fuchun Xu[1], Xiaohong Chen[1], Duanjun Cai[1,], and Junyong Kang[1]*

[1] Fujian Key Laboratory of Semiconductor Materials and Applications, CI Center for OSED, College of Physical Science and Technology, Xiamen University, Xiamen 361005, China

[2] Department of Physics, School of Science, Jimei University, Xiamen 361021, China

[3] These authors contributed equally to this work.

* Email: dcai@xmu.edu.cn

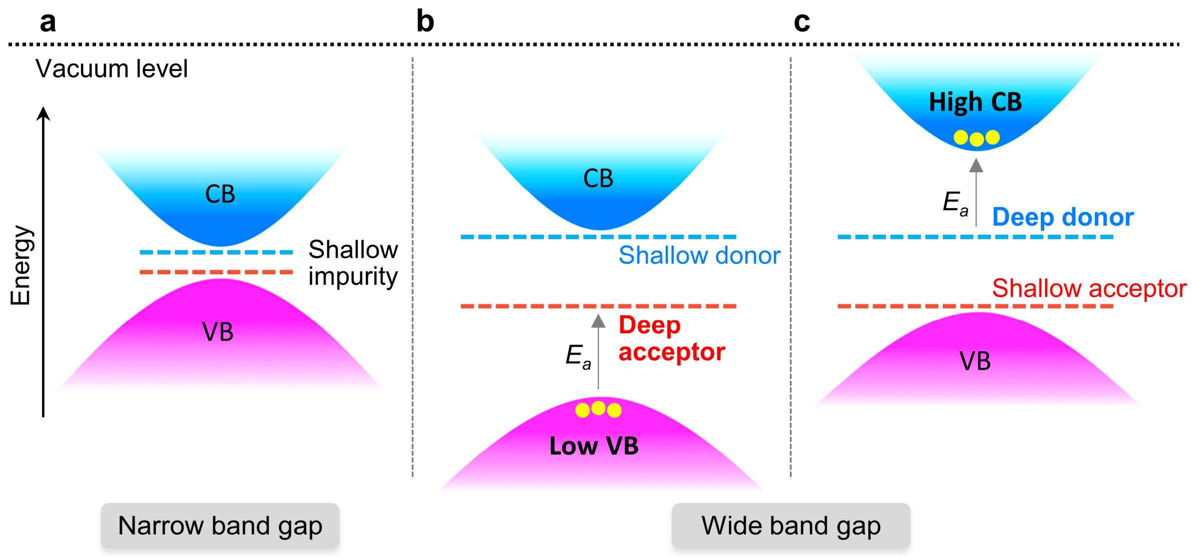

Supplementary Figure 1. Asymmetric doping problem in wide bandgap semiconductors. (**a**) Schematic of the band structure and impurity levels of narrow bandgap semiconductors. **b-c**, Schematic of the band structure and impurity levels of wide bandgap semiconductors with (**b**) low valence band and (**c**) high conduction band.

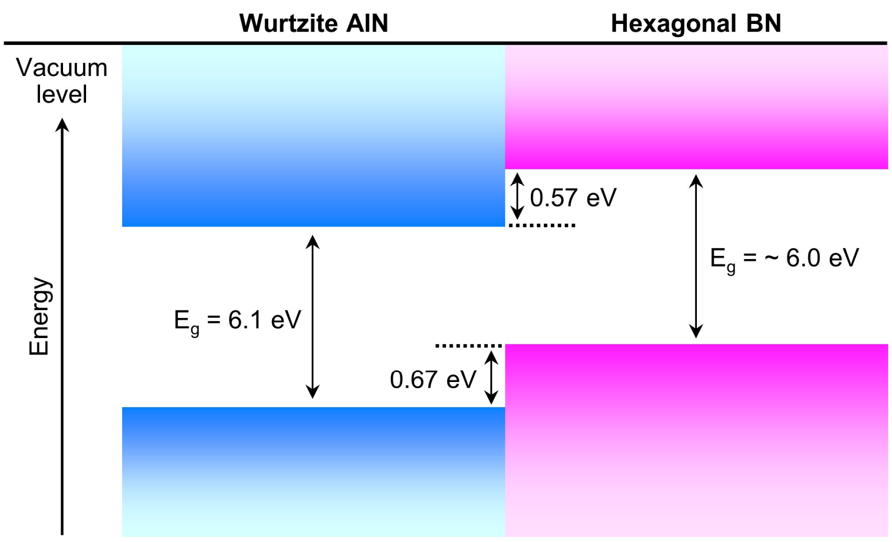

Supplementary Figure 2. Schematic of band alignment of the wurtzite AlN and hexagonal BN.

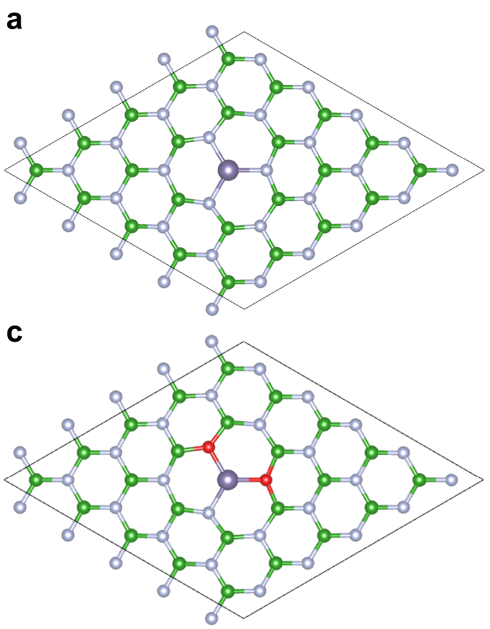

Supplementary Figure 3. Crystal structures of (**a**) h-BN:Ge, (**b**) h-BN:Ge-O, (**c**) h-BN:Ge-O_2, and (**d**) h-BN:Ge-O_3 system, respectively.

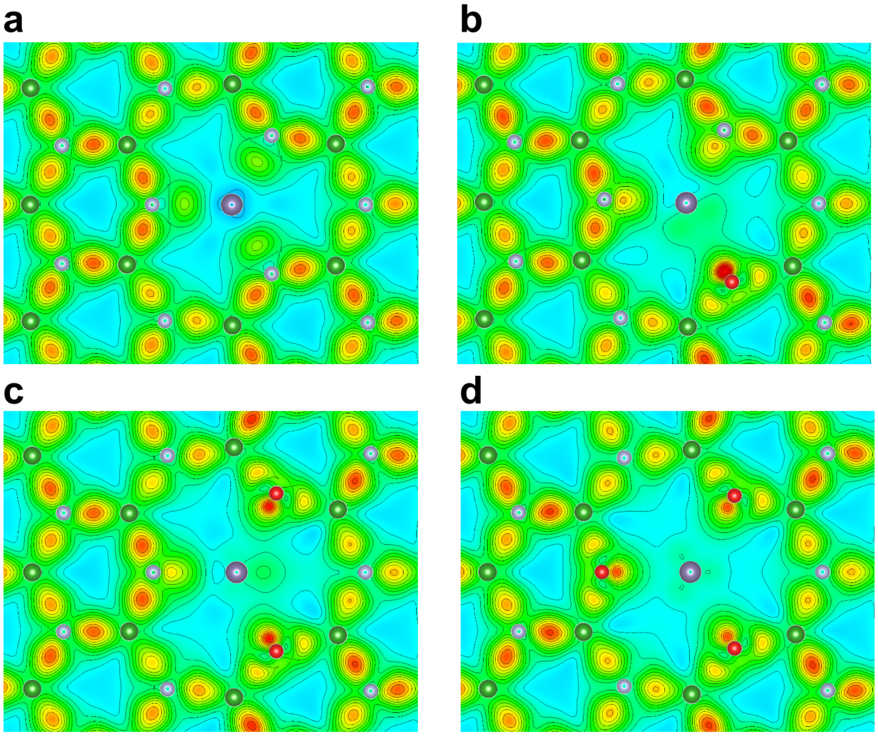

Supplementary Figure 4. Deformation charge density in the plane of (**a**) h-BN:Ge, (**b**) h-BN:Ge-O, (**c**) h-BN:Ge-O_2, and (**d**) h-BN:Ge-O_3 system, respectively.

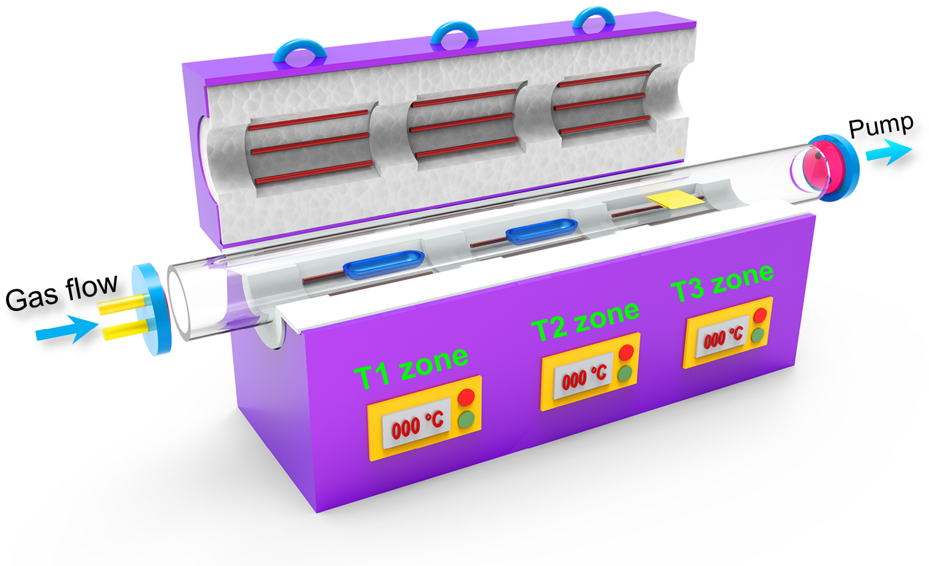

Supplementary Figure 5. Schematic of the LPCVD system for performing the h-BN growth.

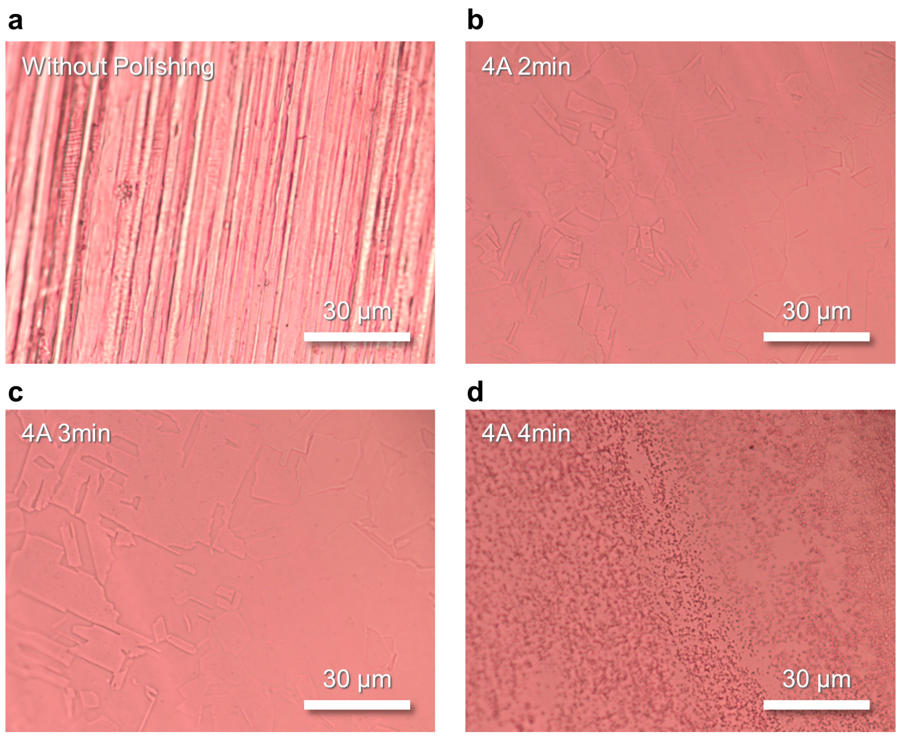

Supplementary Figure 6. Optical images of (**a**) unpolished Cu foil, and polished Cu foils under 4 A current and after (**b**) 2 minutes, (**c**) 3 minutes, and (**d**) 4 minutes of polishing times, respectively.

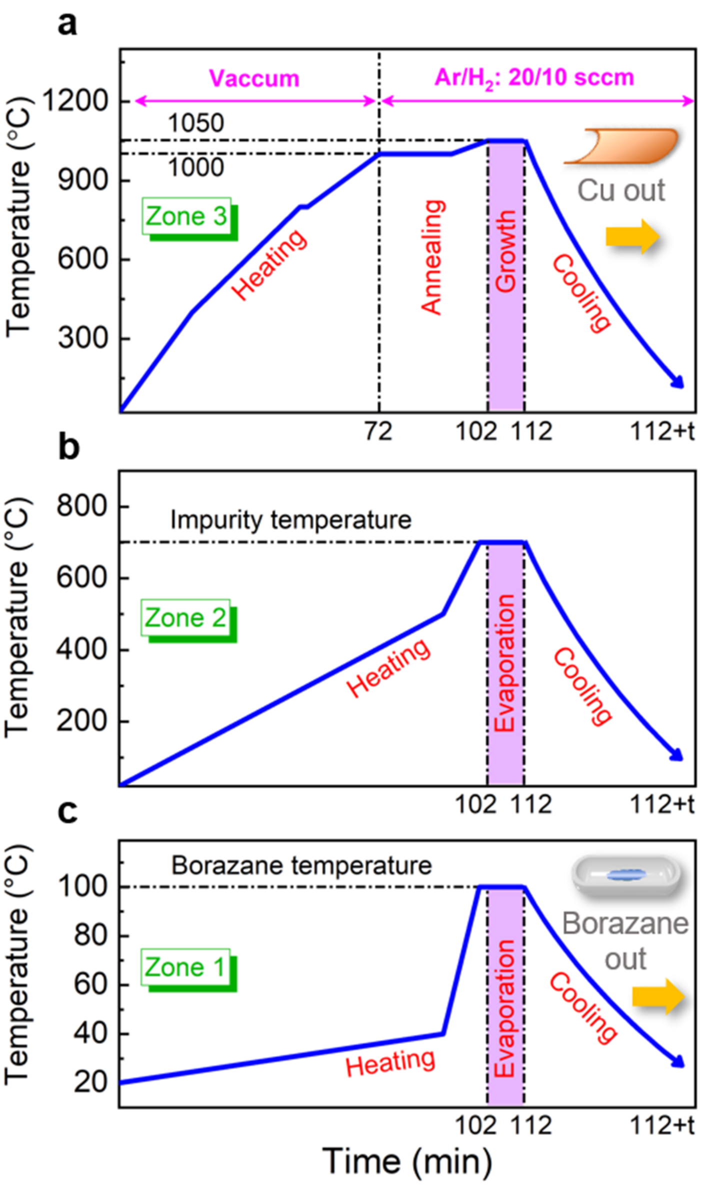

Supplementary Figure 7. Time dependence of experimental parameters in (**a**) Zone 3, (**b**) Zone 2, and (**c**) Zone 1 for growing h-BN films.

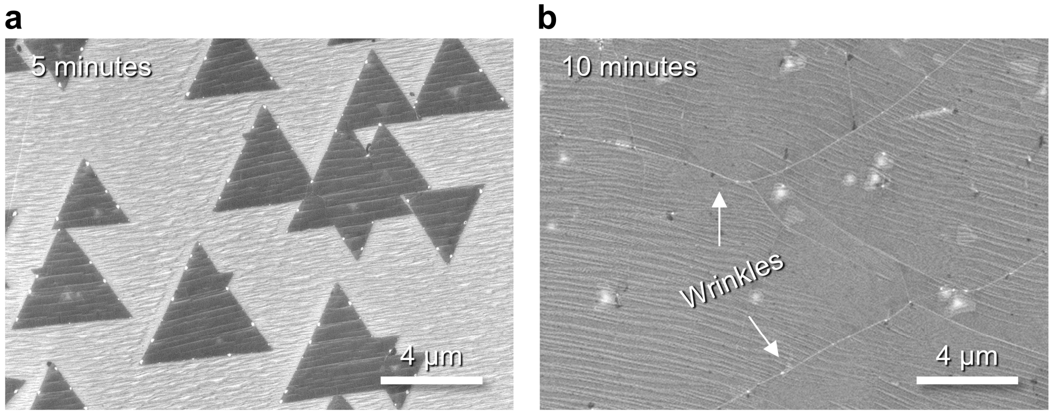

Supplementary Figure 8. (**a**) SEM image of the as-grown undoped h-BN domain using 5 minutes growth time. (**b**) SEM image of the fully coalesced undoped h-BN monolayer using 10 times growth time.

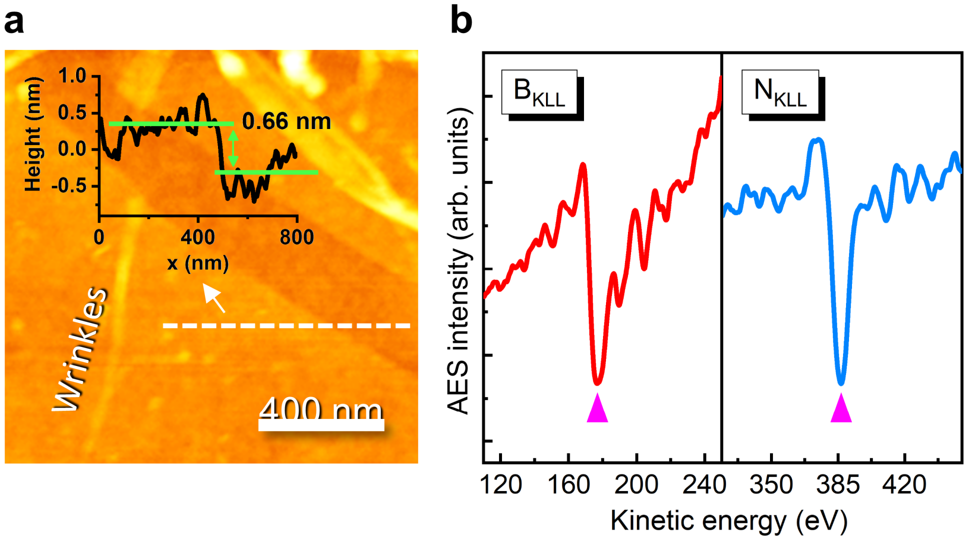

Supplementary Figure 9. (**a**) AFM image of the undoped h-BN monolayer on SiO_2. The thickness of the film is found to be 0.66 nm, as shown in the inset. (**b**) AES spectra of the undoped h-BN film, showing the B_{KLL} and N_{KLL} Auger lines located at 177.1 eV and 385.6 eV, respectively, demonstrating the good quality of h-BN.

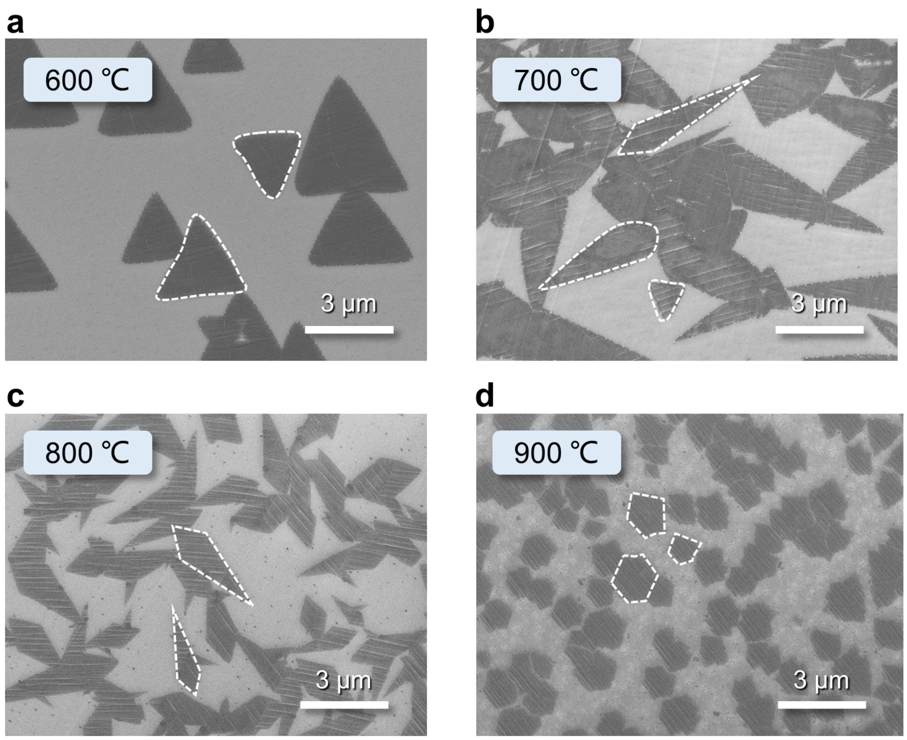

Supplementary Figure 10. (**a**)-(**d**) SEM images of the as-grown h-BN:Ge-O monolayer on Cu foil at various temperatures of Zone 2 after 5 minutes growth times.

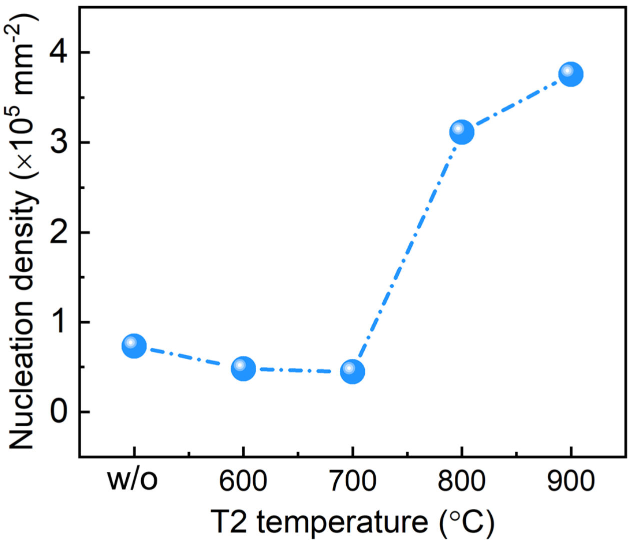

Supplementary Figure 11. The nucleation density of h-BN:Ge-O monolayer at various temperatures of zone 2 after 5 minutes growth times.

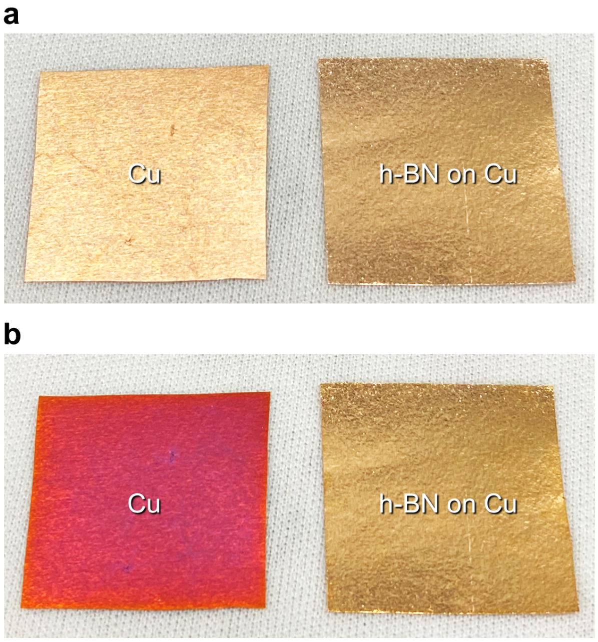

Supplementary Figure 12. Photographs of bare Cu foil and Cu foil with fully coalesced h-BN monolayer (**a**) before and (**b**) after 10 minutes heating at 200 ºC. The Cu foil with h-BN monolayer can withstand high temperature heating.

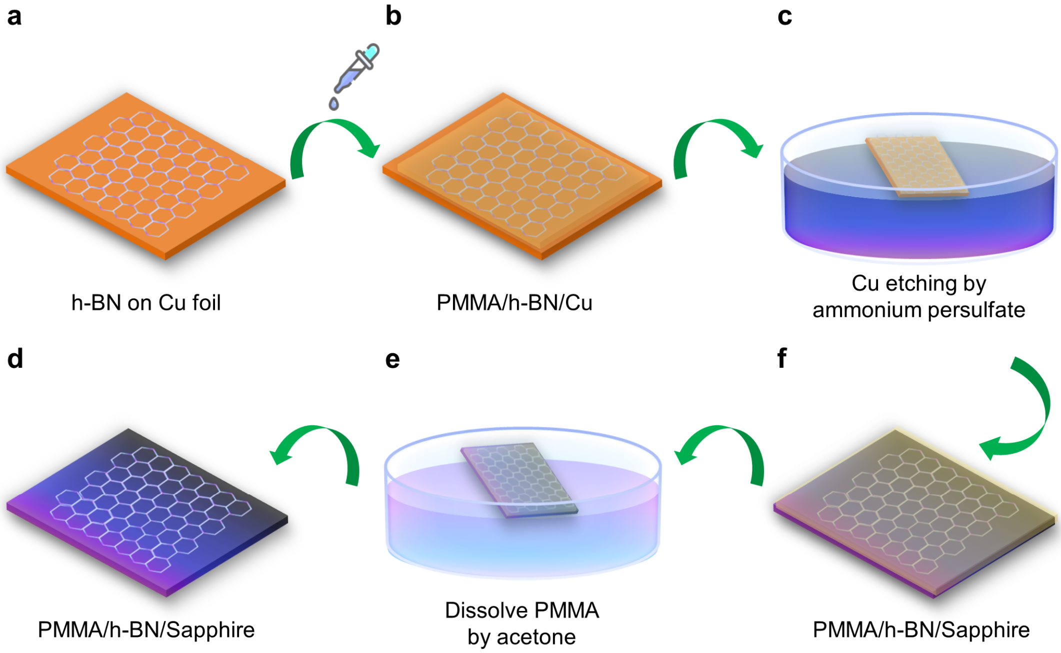

Supplementary Figure 13. (**a**)-(**f**) Schematic of the wafer-scale h-BN transfer process using PMMA-assisted wet transfer method.

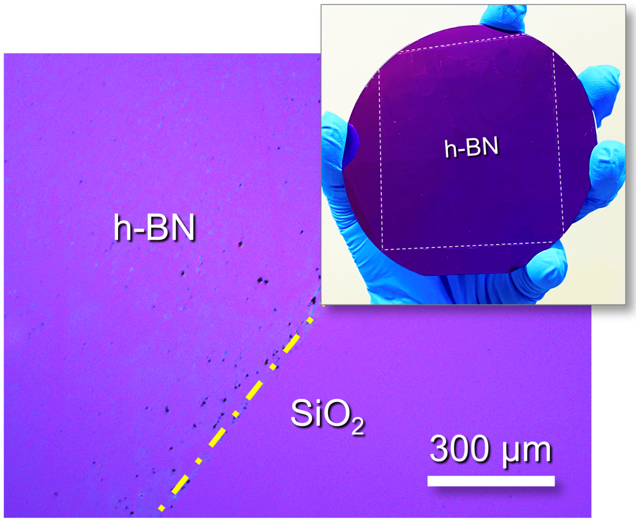

Supplementary Figure 14. Optical image of the h-BN transferred on a 4-inch SiO_2/Si substrate. The inset shows the photograph.

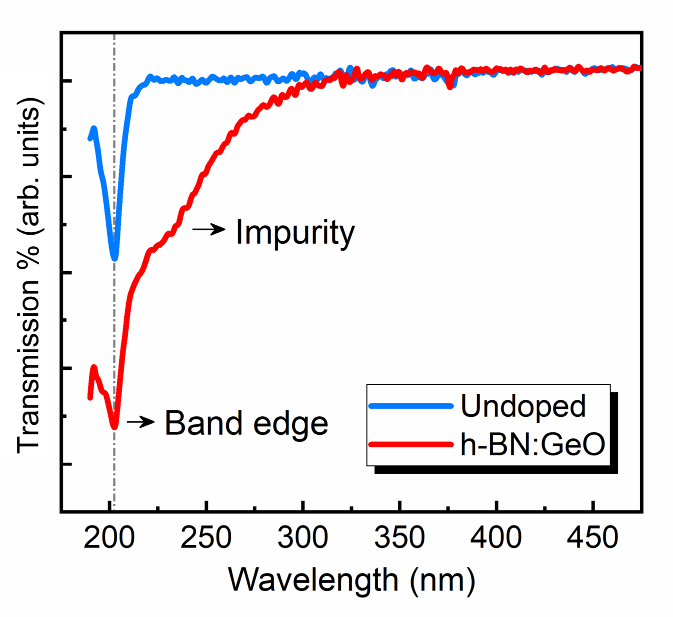

Supplementary Figure 15. Transmission spectra of the undoped h-BN and h-BN:Ge-O monolayer.

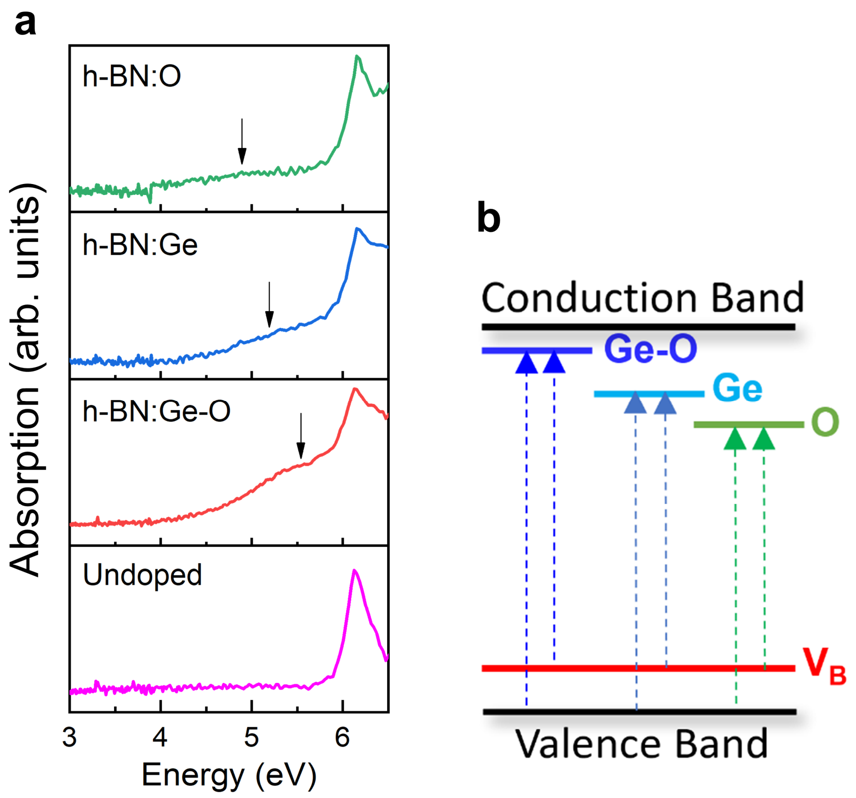

Supplementary Figure 16. (**a**) Absorption spectra of h-BN:O, h-BN:Ge, h-BN:Ge-O, and undoped h-BN films. (**b**) Energy band diagram showing the transitions responsible for the absorption peaks of doped h-BN.

Supplementary Note 1: Origin of the absorption peaks

In order to confirm the origin of the absorption in h-BN:Ge-O, we further measured absorption spectra of h-BN:O, h-BN:Ge, and undoped h-BN samples for comparison, as shown in Figure S16(a). It can be seen that the sharp band-edge absorption peaks of intrinsic h-BN is at 209.6 nm (5.93 eV). The impurity related absorption peaks for h-BN:Ge-O, h-BN:Ge and h-BN:O appear at 5.53 eV, 5.19 eV and 4.89 eV, respectively. These results distinguish the different origins. Detailed examination further shows that the absorption shoulder of h-BN:Ge-O is abroad, covering the near band-edge band down to the h-BN:O related peak. This may mean that in the h-BN:Ge-O sample, the absorptions may include transitions from Ge, O, and Ge-O impurity levels. Of course, the Ge-O type impurity dominates. Due to the acceptor-like state such as boron vacancy may be formed during the high temperature growth, the absorptions may stem from the transitions between the dopant levels (Ge-O, Ge, O) and the acceptor-like/valence band levels, as summarized in Figure S16(b). However, pertinent assignment of transition levels involved in the absorption requires further analysis based on fine structures of low-temperature PL spectra.

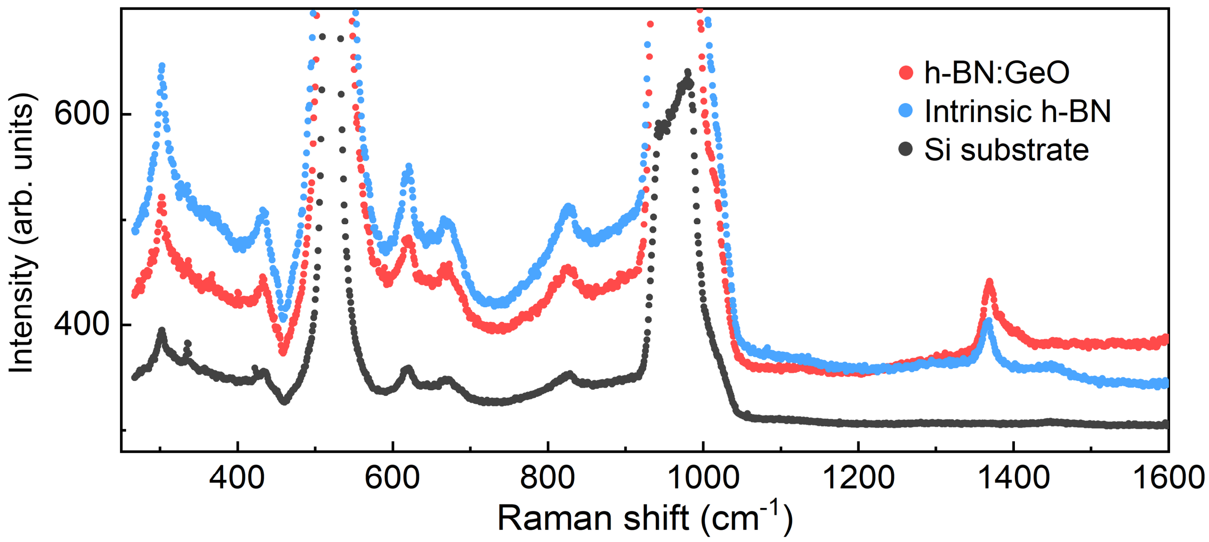

Supplementary Figure 17. Raman spectra of the Si substrate, intrinsic undoped h-BN and h-BN:Ge-O films.

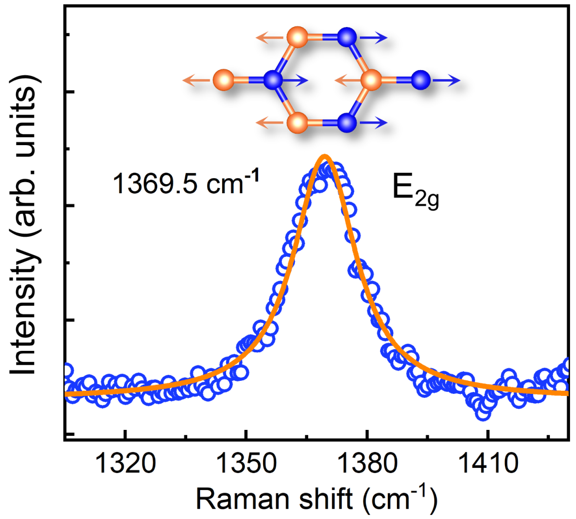

Supplementary Figure 18. Raman spectrum of the undoped h-BN monolayer, showing a typical symmetrical feature. The inset shows the corresponding E_{2g} vibrational mode.

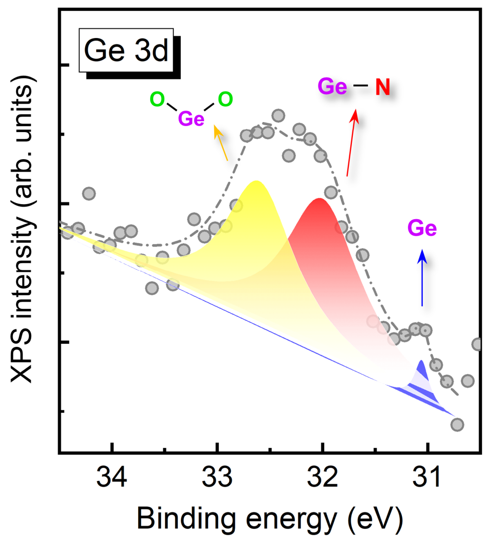

Supplementary Figure 19. XPS spectrum of the h-BN:Ge-O film, showing the Ge 3d core level.

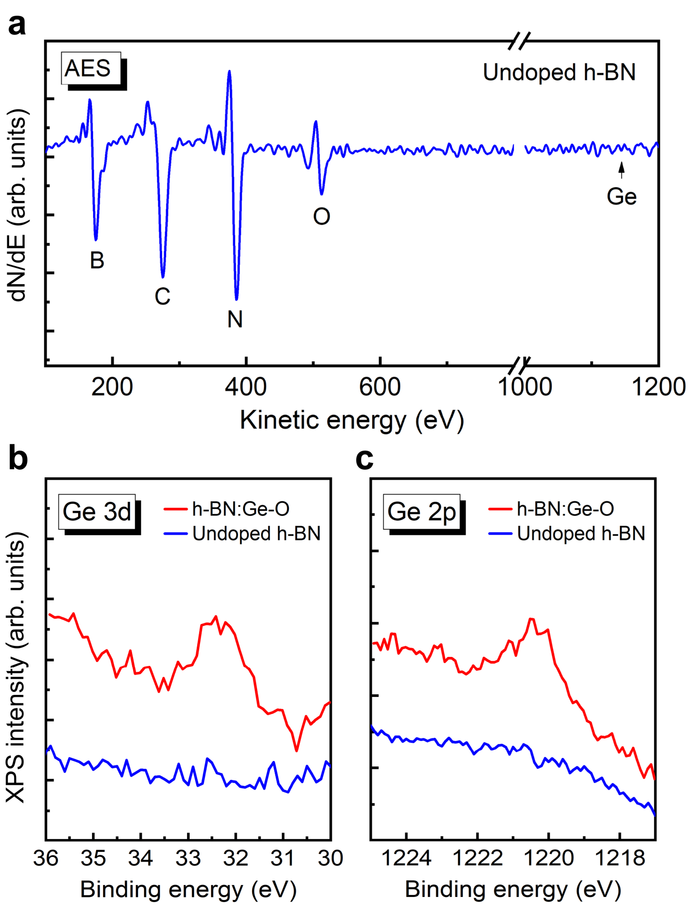

Supplementary Figure 20. (**a**) AES spectrum of the undoped h-BN film. (**b**)-(**c**) XPS spectra of the h-BN:Ge-O and undoped h-BN films, showing the Ge 3*d* level (**b**) and Ge 2*p* level (**c**).

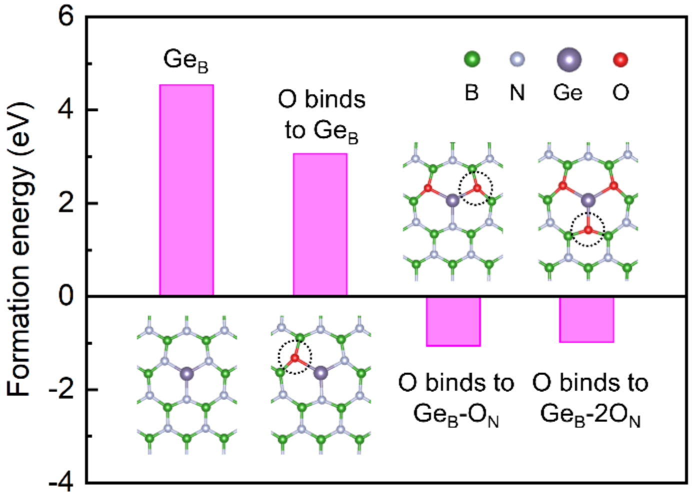

Supplementary Figure 21. Formation energies of Ge_B, Ge_B-O_N, Ge_B-2O_N, and Ge_B-3O_N in h-BN.

Supplementary Note 2: Driving force of O binding to Ge donor

We further performed first-principle calculations on the formation energies of individual Ge_B impurity, Ge_B-O_N dimer, and Ge_B-2O_N trimer in h-BN monolayer to investigate the driving force of O binding. The formation energies of impurity *X* is defined as follow:[1]

$$E^f[X] = E_{tot}[X] - E_{tot}[original] - \sum_i n_i \mu_i \, ,$$

where $E_{tot}[X]$ is the total energy derived from the system with impurity *X* doping, $E_{tot}[original]$ is the total energy of the system before the impurity *X* doping, n_i indicates the number of atoms of type i that have been added (n_i >0) or removed (n_i <0) from the supercell when the impurity is formed, and the μ_i are the chemical potentials of these species. It is interesting to see that the O binding will lower the formation energy of Ge or Ge-O impurities. The decreasing formation energy follows the trend: Ge_B > Ge_B-O_N > Ge_B-2O_N > Ge_B-3O_N. This implies that the binding and coupling with O could minimized the formation energy of Ge, which is the driving force for the formation preference of Ge-O bonds. More importantly, the formation energies of single O binding to Ge_B-O_N dimer, and O binding to Ge_B-2O_N trimer are reduced to -1.06 eV and -0.98 eV, respectively. The negative formation energies indicate that the Ge-O_2 and Ge-O_3 are most likely to form during the growth. These simulation results strongly prove that there is a driving force that prompts the O binding to the Ge donor. Therefore, not only for the statistics, the Ge-O_2 trimer and Ge-O_3 tetramer doping are indeed favorable in the thermodynamics aspect. On the other hand, in the experiments, Ge_2O_3 was employed as the precursor for Ge-O doping, which actually already have the Ge-O binding before incorporation into the h-BN.

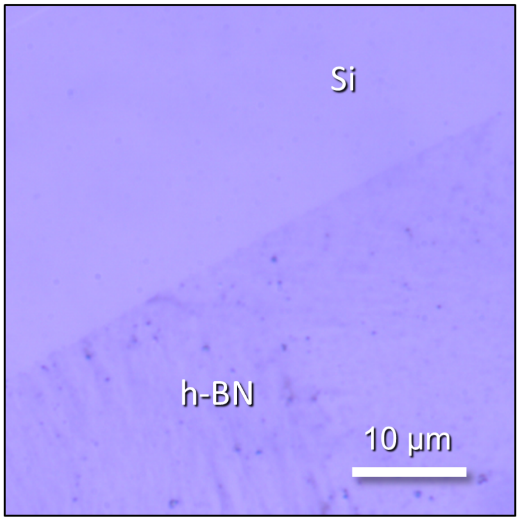

Supplementary Figure 22. Optical image of the h-BN film transferred on a Si substrate for CAFM test.

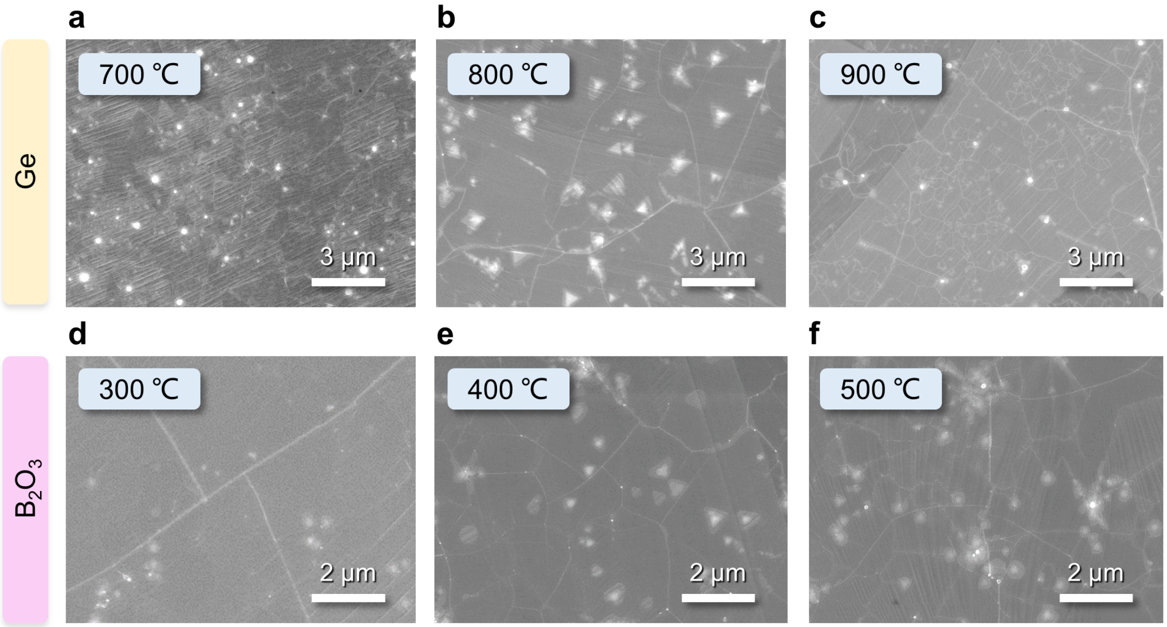

Supplementary Figure 23. (**a**)-(**c**) SEM images of the as-grown h-BN:Ge monolayer using Ge powder as impurity precursor at various T2 temperature. (**d**)-(**f**) SEM images of the as-grown h-BN:O monolayer using B_2O_3 powder as impurity precursor at various T2 temperature.

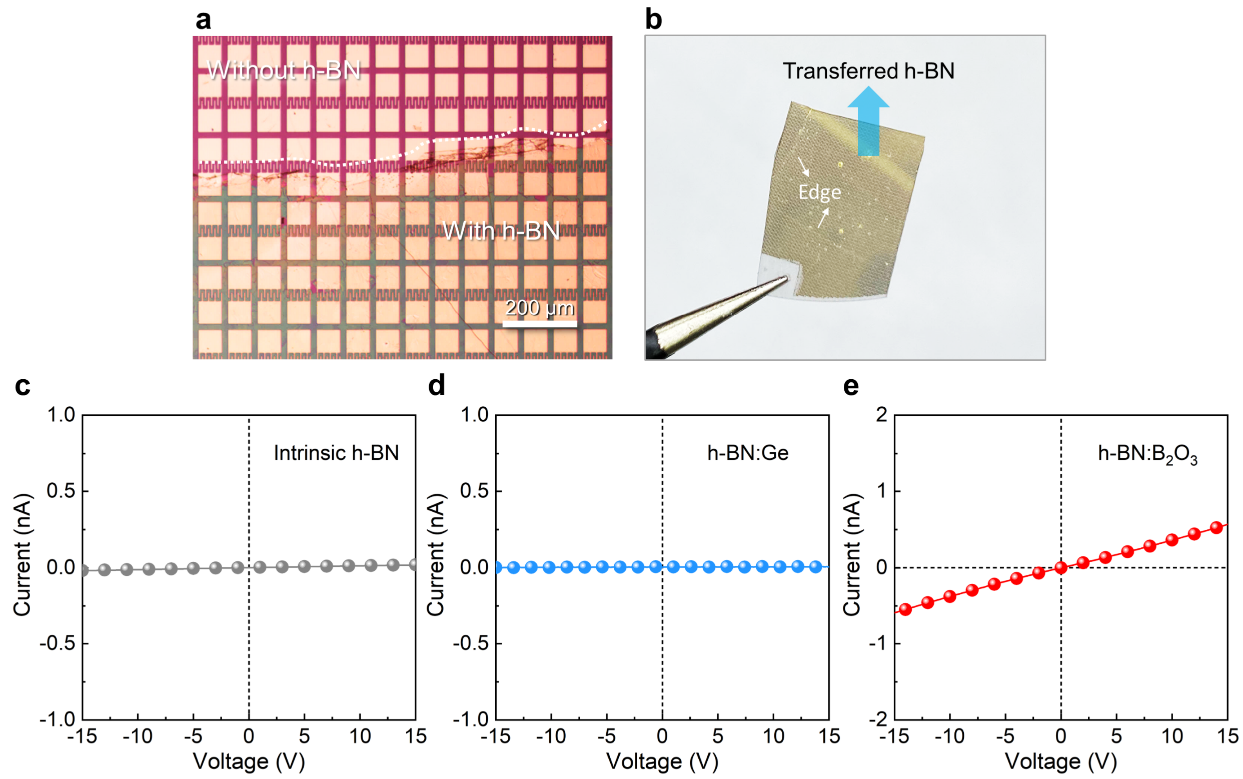

Supplementary Figure 24. (**a**) Optical image and (**b**) photograph of h-BN monolayer transferred on sapphire substrate with Au electrode array. I-V curves of (**c**) intrinsic h-BN, (**d**) h-BN:Ge, and (**e**) h-BN:B_2O_3 film, respectively.

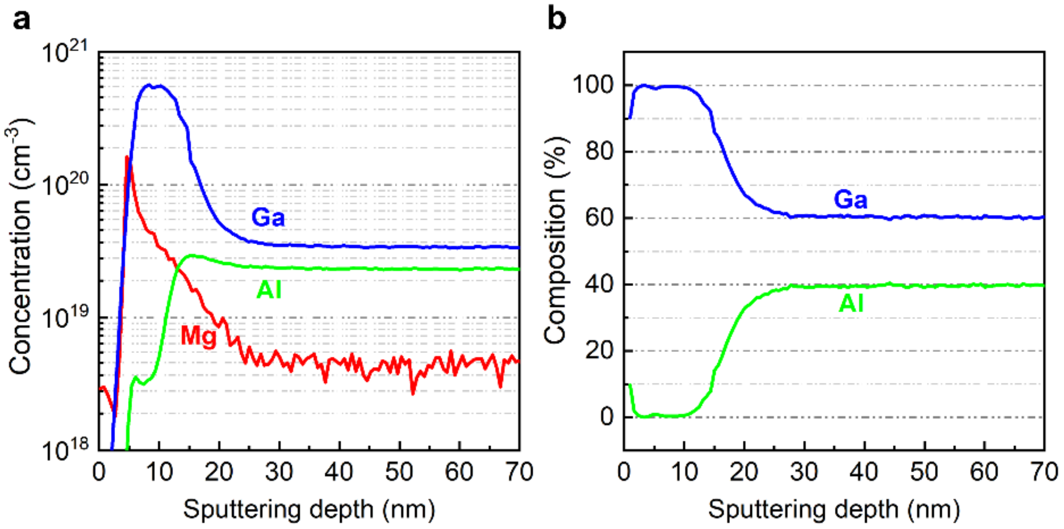

Supplementary Figure 25. (**a**) Element concentration and (**b**) element composition of the *p*-GaN/AlGaN epilayer.

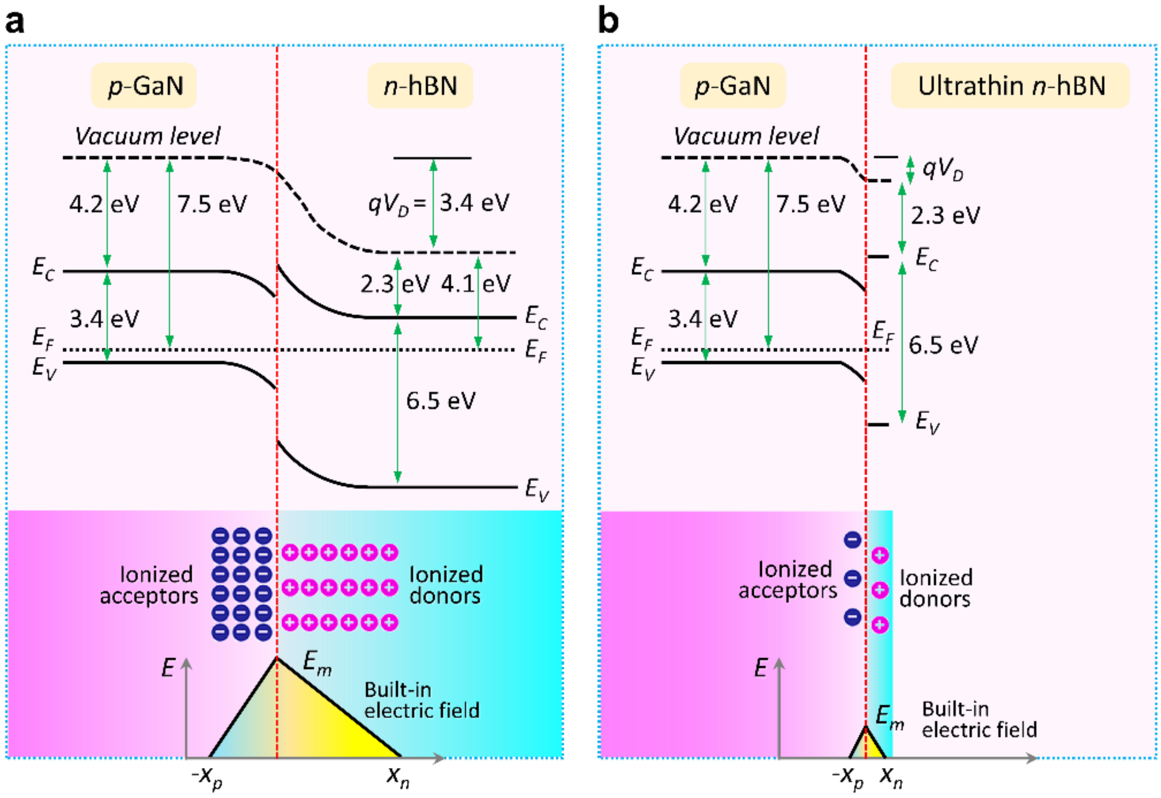

Supplementary Figure 26. Energy band diagram, space charge distribution, and electron field distribution of *p*-GaN/*n*-hBN junction in thermal equilibrium with (**a**) thick h-BN layer and (**b**) ultrathin h-BN layer.

Supplementary Note 3: Energy band diagram, space charge and electric field distribution of the p-n junction

The doping level and carrier density of *p*-GaN should be larger than those of *n*-type h-BN layer. This may impact the diode behavior. The energy band diagram, space charge distribution, and electric field distribution in this *p*-GaN/*n*-hBN junction in thermal equilibrium are shown in Figure S26. For a thick *n*-hBN layer, the *n*-hBN layer is not completely depleted (a) whereas the ultrathin *n*-hBN should be largely depleted (b). From the electric field distribution in Figure S26, we can see that he built-in electric field E_m at the depleted ultrathin h-BN interface will decrease but still work for the typical diode behavior. This decreasing built-in field with the decreasing h-BN thickness could lead to decreasing the forward turn-on voltage, which has been confirmed by the I-V test, as shown in Fig. 5(d)-(e). Therefore, the forward turn-on voltage for the monolayer h-BN case (1.44 eV) is smaller than that the 6-layer *n*-hBN case (3.11 eV).

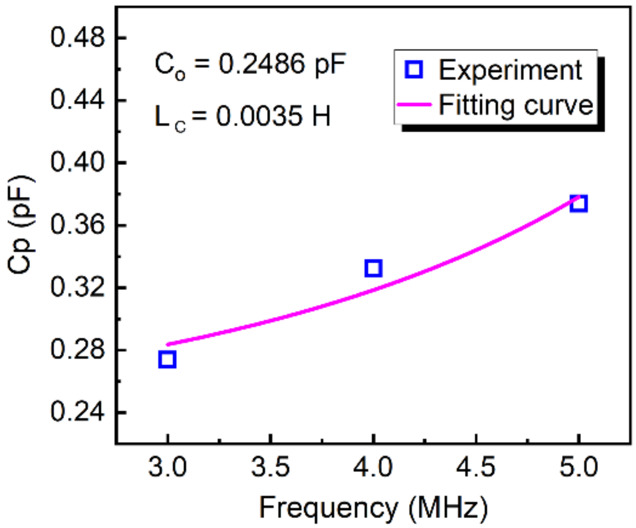

Supplementary Figure 27. Effective capacitance as a function of frequency and the fitting curve of the *n*-hBN/*p*-GaN junction under -2 V.

Supplementary Note 4: Effective capacitance vs applied frequencies

The phenomenon of increasing capacitance with the increasing frequency is unusual for a *p*-*n* junction. Generally, the capacitance should be constant over frequency. But when the applied frequencies approach the capacitor's self-resonant frequency, a parasitic series inductance will work and result in an effective capacitance (C_E) that is larger than the nominal capacitance (C_O).[2,3] The effective capacitance can be described as:

$$C_E = \frac{C_O}{1-(2\pi f)^2 L_C C_O},$$

where f is the applied frequency, L_C is the parasitic series inductance of the capacitor. In practice the inductance of the capacitors is rather small so that $C_E = C_O$. When the inductance becomes considerable, at high frequencies the effective capacitance will increase along with the frequency. Thus, we can find out that the origin of the capacitance increasing in this *n*-hBN/*p*-GaN junction should be attributed to the considerable inner series inductance. By fitting the C_p-f data with above equation, as shown in Figure S27, we can obtain the value of the inner series inductance. As a result, the nominal capacitance C_O is 0.2486 pF and the inductance L_C is 0.0035 H. This considerable inner inductance in the *n*-hBN/*p*-GaN junction should be highly related with the unique 2D layered structure of the multilayer h-BN. Because of the weak van der Waals interaction between *n*-h-BN layers, the possible formation of multiple conducting micro-channels could be the reason to introduce this inner inductance. However, reliable evidences and explanations need further thorough investigations.

Supplementary method about the FET device

Instead of Hall measurement, we obtained further electrical properties of the n-type h-BN monolayer by the FET device method.[4] The resistivity ρ of n-type h-BN monolayer was calculated be about 2.29×10^4 Ω cm from the I_{sd} - V_{sd} curve at V_g = 0 (Fig. 4g-i). In addition, the channel conductance g_m of a FET device can be calculated as follow:

$$g_m = \frac{dI_{sd}}{dV_g} = \frac{W}{L}\mu_e C_g V_{sd},$$

where W and L are the width and length of the channel, μ_e is the electron mobility, C_g is the gate capacitance. For SiO_2, the gate capacitance is:

$$C_g = \frac{\varepsilon_0 \varepsilon_r}{d},$$

where ε_0 is the vacuum permittivity, ε_r is the relative dielectric constant of gate SiO_2 (3.9), and d is the thickness of gate SiO_2 layer. According to these two equations, the electron mobility of n-type h-BN was calculated to be 0.014 cm^2 V^{-1} s^{-1} as follow:

$$\mu_e = \frac{dI_{sd}}{dV_g}\frac{L}{W}\frac{d}{\varepsilon_0 \varepsilon_r V_{sd}}.$$

The electron mobility of n-type h-BN monolayer is relatively small, which can be contributed to the interaction and scattering effects from the SiO_2 substrate. The SiO_2 substrate is not smooth sufficiently and there are many dangling bonds exist on the surface. Many works have reported that the SiO_2 substrate may deteriorate the mobility of 2D materials.[5,6] The relation between the resistivity, conductivity, and carrier concentration can be defined as following equation:

$$\rho = \frac{1}{\sigma} = \frac{1}{nq\mu}.$$

Therefore, the electron concentration n can be calculated from:

$$n = \frac{1}{\rho q\mu}.$$

According to above equation, the electron concentration of n-type h-BN monolayer is determined to be 1.94×10^{16} cm^{-3}.

Supplementary References

1. Freysoldt, C. *et al.* First-principles calculations for point defects in solids. *Rev. Mod. Phys.* **86**, 253–305 (2014).

2. Klionskii, M. D. & Tsiklauri, G. N. Determining the value of reference air capacitors at high frequencies. *Meas. Tech.* **15**, 1046–1049 (1972).

3. Gulyaev, M. A. Measurement of inductance and effective capacitance of a standard air capacitor. *Meas. Tech.* **6**, 758–761 (1963).

4. Tang, Y. *et al.* Tunable p-Type Conductivity and Transport Properties of AlN Nanowires via Mg Doping. *ACS Nano* **5**, 3591–3598 (2011).

5. Dean, C. R. *et al.* Boron nitride substrates for high-quality graphene electronics. *Nat. Nanotechnol.* **5**, 722–726 (2010).

6. Xue, J. *et al.* Scanning tunnelling microscopy and spectroscopy of ultra-flat graphene on hexagonal boron nitride. *Nat. Mater.* **10**, 282–285 (2011).
